# Global and targeted approaches to single-cell transcriptome characterization

**DOI:** 10.1093/bfgp/elx025

**Published:** 2017-09-25

**Authors:** Aleksandra A Kolodziejczyk, Tapio Lönnberg

**Affiliations:** 1 Wellcome Trust Sanger Institute, Wellcome Genome Campus, Hinxton, Cambridge, UK; 2 EMBL-European Bioinformatics Institute, Wellcome Genome Campus, Hinxton, Cambridge, UK

**Keywords:** single-cell, heterogeneity, transcriptomics, RNA-seq, RNA sequencing, microarrays, RT-qPCR

## Abstract

Analysing transcriptomes of cell populations is a standard molecular biology approach to understand how cells function. Recent methodological development has allowed performing similar experiments on single cells. This has opened up the possibility to examine samples with limited cell number, such as cells of the early embryo, and to obtain an understanding of heterogeneity within populations such as blood cell types or neurons. There are two major approaches for single-cell transcriptome analysis: quantitative reverse transcription PCR (RT-qPCR) on a limited number of genes of interest, or more global approaches targeting entire transcriptomes using RNA sequencing. RT-qPCR is sensitive, fast and arguably more straightforward, while whole-transcriptome approaches offer an unbiased perspective on a cell’s expression status.

## Why is single-cell transcriptomics useful?

Transcriptomics, defined as high-throughput quantitative study of the total complement of cellular RNA (or more narrowly mRNA) molecules, is a powerful and widely used approach for describing states of cellular activity. This includes dynamic changes in cell state during development and differentiation, and responses to environmental or experimental perturbations. While the quantity of mRNA is not the only determinant of expression and activity of the encoded protein, it provides a highly usable proxy. Therefore, transcriptomics often represents the most efficient means for defining cellular states and studying phenotypic changes and the underlying signalling networks.

**Figure 1.**
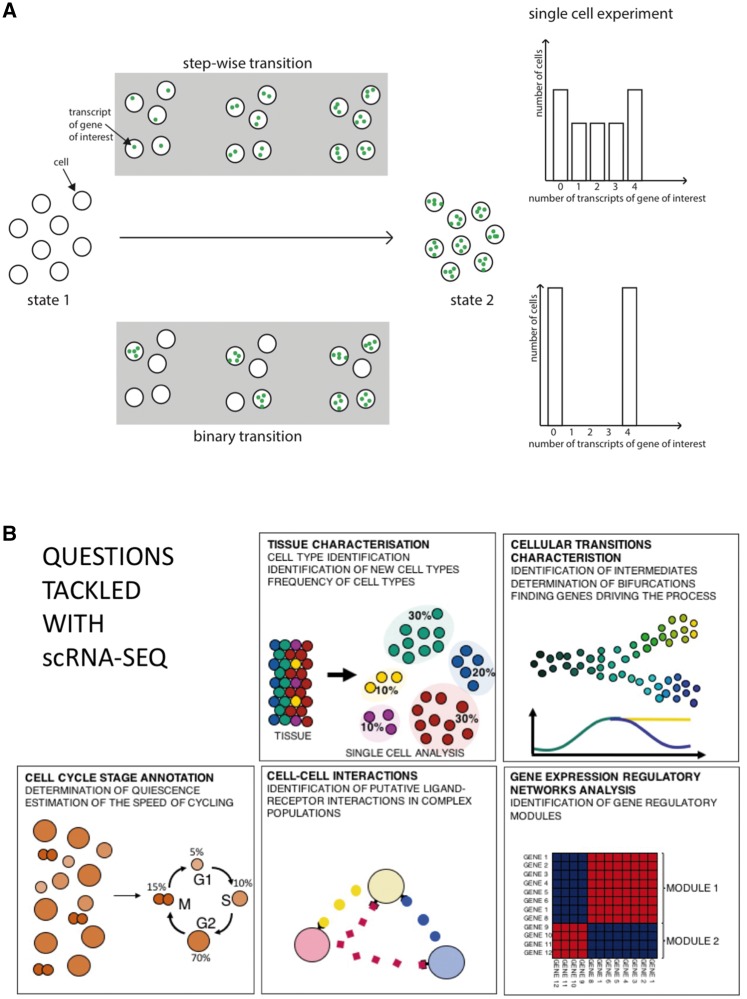
Single-cell methods provide insight into the nature of a population, its subpopulation structure and heterogeneity. (**A**) A conceptual example is the switch of cells from State 1 to State 2 in this schematic diagram. This process could be either a binary or gradual switch in transcriptomic state. While population methods cannot distinguish between the two states, single-cell methods can discriminate between these two transitions. (**B**) Examples of biological questions addressed with single-cell RNA sequencing (scRNA-seq).

Transcriptomics techniques, such as microarrays and massively parallel sequencing, are typically applied on samples consisting of thousands or millions of cells. This implies an assumption that phenotypically similar cells in a population are similar also in terms of molecular composition, and are thus represented with reasonable accuracy by the average values of the population. However, a growing body of data contradicts this assumption [1–5]. In fact, the emerging view strongly suggests that transcriptomes of even closely related cells exhibit considerable heterogeneity. Conceptually, the biological heterogeneity can be divided into (1) heterogeneity originating from stochastic nature of biochemical processes including gene expression, (2) heterogeneity originating from slightly different molecular microenvironments and different signalling histories of each cell and (3) population heterogeneity, which is deterministic and ‘hard-wired’ causing subsets of cells intrinsically to express different properties (Figure 1A) [6].

Cell-intrinsic and environmental factors contributing to this heterogeneity are incompletely understood, as are its full biological consequences. In an experimental setting, such variation may arise from asynchronous stages of cell cycle [7–11], uneven partitioning of molecules during cell divisions and differences in cellular signalling histories or epigenetic modifications before the experiment in question. Moreover, transcription of both prokaryotic and eukaryotic genes has been documented to frequently follow stochastic burst-like kinetic patterns, with relatively short but intense bursts of transcription being followed by longer inactive periods during which mRNA levels decay [4, 12–14]. Recent studies suggest that such bursting is widespread, although the duration of the bursts and intervals can vary considerably [15]. Mechanistically, expression bursts are dependent on the stochastic processes of transcription factors and RNA polymerase binding [16]. In line with this intrinsic stochasticity, single-cell gene expression data typically follows negative binomial distribution [17]. An important implication of this is that the ubiquitously used population-wide average values are not accurate representations of the typical single cell. In terms of understanding the basic biology of gene expression and the structure of a cell population, these reasons make a strong argument for performing transcriptomics analyses at the single-cell level, and highlight the need for developing robust system-wide methods.

Single-cell efforts have also been further motivated by the innumerable potential applications involved (Figure 1B). An obvious benefit is the possibility to study rare types of cells, either too limited in number or too sparsely distributed for conventional bulk transcriptomics [18]. Important examples include early stages of embryonic development [19] and circulating cancer cells [20, 21]. Another important issue that can be potentially addressed by single-cell analysis is tissue heterogeneity. Many biological systems of high medical significance, such as hematopoietic lineages [22] and neural cells [23], are composed of intermixed differentiated cell types acting in coordination but using different molecular pathways. The response of such a population to a perturbation is likely to be profoundly mixed, and thus data obtained from bulk methods most certainly blend true single-cell transcriptomes and hence will be challenging to interpret [24, 25]. For example, only selected subsets of blood cells are likely to react to a vaccine, or cells of heterogeneous tumours can display widely different responses to a drug. With single-cell transcriptomics, such complex population structures can be dissected and cells of interest can be studied without the confounding effects of population-level averaging. Importantly, resolving heterogeneous populations potentially provides valuable information about transitions between distinct developmental or activation states. By identification of cells in transitional intermediate states one can infer order of regulatory events leading to cellular state transitions. Thus, while single-cell transcriptomics is still in many ways a relatively immature field of research in a state of rapid development, it is already proving its potential and a multitude of research and diagnostic applications are likely to follow.

## RT-qPCR is a sensitive method for targeted analysis of genes of interest

The initial and still widely used way of studying gene expression in single cells is by quantitative reverse transcription PCR (RT-qPCR). Its sensitivity, precision, reproducibility and wide dynamic range has made it a tool of choice for studying mRNA expression and validating findings from high-throughput studies, in particular microarrays. In addition to widespread use in research, numerous diagnostic applications of RT-qPCR have been developed [26]. The mainstream RT-qPCR strategies are based on real-time optical monitoring of complementary DNA (cDNA) amplification using either intercalating dyes [27] or fluorescing hydrolysis probes [28]. As the PCR reaction is intrinsically scalable, in suitable conditions it allows amplification from even single-cell quantities, which was demonstrated early for both DNA and cDNA templates [29, 30]. Accordingly, the standard RT-qPCR workflow is conceptually applicable to single-cell material without profound modifications.

A key consideration in these single-cell applications is prevention of loss of RNA, leading to requirement for so-called single-tube protocols where cell lysis, reverse transcription and PCR are performed without intervening purification steps. This is made possible by low final concentrations of sample-derived RNase and potential inhibitory molecules such as salts, urea, heparin or immunoglobulins [31–33], which in bulk studies typically require depletion by a dedicated purification, precipitation or extraction process. The buffers of the subsequent reaction steps, including lysis, are designed to be compatible and enzymes used in the previous step are inactivated by heat treatment. The unforgivingly low amount of starting material also sets high demands on RT efficiency, although absolute efficiency is also gene-dependent [34, 35]. Overall, many of the technical considerations and pitfalls are in common with bulk RT-qPCR assays and include template quality, standardization of the RT reaction and assay design [31, 36]. The recently proposed the minimum information for publication of quantitative real-time PCR experiments (MIQE) guidelines serve to draw attention to these critical and often neglected issues and should also be taken into account in single-cell studies [37].

Unlike microarrays or RNA sequencing (RNA-seq), single-cell RT-qPCR has the potential for detecting transcripts without a preamplification step (Table 1). Theoretically, single molecules can be detected, although reproducible quantification has been reported to require ∼≥20 copies per cell, thus limiting the analysis to intermediate to high copy number mRNAs [32]. Furthermore, without preamplification only a few (∼≤10) genes can be measured simultaneously, as parallel assays require aliquoting of the sample [33]. Taniguchi *et al*. [35] have proposed a bead-based strategy for immobilizing and reusing cDNA molecules, thus overcoming the need of aliquoting the sample. However, the number of possible sequential assays from a single cDNA library remains relatively small, theoretical output also being limited by instrument time.

**Table 1.** Comparison of approaches for single-cell transcriptome characterization

Property	RT-qPCR	Microarrays	RNA-seq
Genes analysed	• Hundreds	• Thousands	• All
	• All genes	• Only genes with poly(A)	• Only genes with poly(A)
	• Knowledge-driven choice of genes	• Knowledge-driven choice of genes	• Unbiased
Alternative splicing information	• Yes, but challenging	• Yes, knowledge driven	• Yes, unbiased
Biases	• Biases from preamplification (if used)	• Biases from preamplification	• Biases from preamplification
	• Biases caused by detection method (dye, Taqman probes, etc.)		
		• False negatives for probes at 5′ end of long genes	
Sensitivity	• High	• No detection of low copy number transcripts	• No detection of low copy number transcripts
Multiplexing	• 96 cells×96 genes with the Biomark^TM^	• No	• From 96 cells (C1^TM^) to thousands of cells (Chromium^TM^)
Quantification	• Relative (absolute possible with spike-ins)	• Relative	• Absolute
Data obtained	• Cq values	• Intensities	• Read counts, RPKM/FPKM/TPM
Data analysis	• Standard univariate or multivariate statistics	• Standard microarray data analysis pipelines	• Standard RNA-seq data analysis pipelines
			+ Bespoke methods

FPKM, fragments per kilobase per million; RPKM, reads per kilobase per million; TPM, transcripts per million.

The possibilities of single-cell RT-qPCR can be significantly extended by methods allowing quantitative preamplification of mRNAs independent of gene sequence or transcript size. These protocols are typically based on the use of poly-dT primers and exploit either exponential PCR amplification or *in vitro* transcription (IVT)-based linear amplification [38]. Thus, a single cell can provide a virtually infinite supply of cDNA, making the availability of suitable RT-qPCR assays and relatively high running costs the limiting factors for sample throughput. However, amplification also leads to increased noise and can introduce biases and should therefore not be used without appropriate quality control. Allowing more extensive multiplexing and thus more powerful experimental designs, preamplification has become a widely used routine step in single-cell RT-qPCR studies [39–41]. Nevertheless, multiplexing approaches are ultimately limited by the amount of manual work involved as well as assay costs. To overcome these limitations, microfluidics-based multiplex assay platforms have been developed. These include the Biomark^TM^ Dynamic Arrays (Fluidigm), using which 96 samples can be interrogated with 96 parallel primer–probe assays [42]. A key promise of such tools is the potential to uncover novel regulatory relationships between the genes under investigation [43, 44].

A common pitfall in RT-qPCR workflows is presented by data processing and in particular normalization. The purpose of normalization is to eliminate bias resulting from differences in cDNA amounts between samples, associated with unequal loading of starting material, or unequal losses during sample processing. In single-cell experiments, differences in cell size present an important additional consideration. The functional activity of mRNAs is ultimately determined by their intracellular concentration rather than absolute copy number [45]. Thus, including a normalization step for cell size might improve the biological value of the analysis, especially if the analysed cells are particularly heterogeneous in size. On the other hand, inappropriate choice of normalization strategy, based on subjective or otherwise incorrect assumptions, can lead to biased or downright erroneous results. These considerations are therefore extremely important in single-cell analysis.

The primary output of an RT-qPCR assay is the number of PCR cycles required to reach a predefined level of signal, herein referred as quantification cycle (Cq), other commonly used synonyms, coined by various instrument manufacturers, being threshold cycle (Ct), crossing point (Cp) and take-off point (TOP). In bulk RT-qPCR studies, normalization is most commonly performed by comparing the measured Cq values with the corresponding values from so-called reference genes, the expression level of which is assumed to be constant within the particular experimental model. The selection of such genes should thus be well justified and preferentially validated by statistical measures. If possible, multiple reference genes should be used. However, at the single-cell level, the usability of the reference gene approach is limited by the ubiquitous cell-to-cell variability in gene expression, extending to traditional reference genes such as *Actb* [46], *Gapdh* [45] and *Tbp* [35]. Nevertheless, in both yeast and mice, many housekeeping genes have been found to be constitutively expressed at a high level with a less than average degree of variability [47–49].

Of note, single-cell experiments provide an intrinsic means for normalization, as the number of cells is constant, i.e. one. While this strategy does not take into account the variability related to differences in cell size, it theoretically allows the measured Cq values to be transformed into mRNA copy numbers per cell. However, as this is based on the assumption of 100% efficiency in reverse transcription and PCR reactions, in practice, the Cq data represent the lowest estimate of the possible true copy number in the cell. Importantly, if the limit of detection for a given experiment is known, for any assay with Cq values exceeding that limit, the copy number can be confidently determined as zero. This is a significant conceptual difference to bulk RT-qPCR studies, wherein such measurements are commonly dismissed as missing values. The limit of detection can be determined by addition of external RNA or cDNA standards to each sample during the lysis step. As such, spike-in standards do not control for pre-lysis variability, and even more rigorous normalization could potentially be achieved by use of standards directly injected into the cells.

With the possibility to measure absence of mRNA species, and in keeping with the model of stochastic burst-like gene expression, multiplexed single-cell RT-qPCR data frequently contain a high proportion of cells with no mRNAs detected [50]. Importantly, the detection frequency of an mRNA correlates with the overall population abundance of the transcript, and hence in such cases can be used as a measure for population-level average expression [33]. Another consideration following from the stochastic nature of gene expression is that at the single-cell level, biological variability (noise) is significantly greater than the technical variability of the RT-qPCR methods. Thus, unlike with bulk RNA-seq, resources will in general be better used by maximizing the number of analysed cells instead of performing technical replicates. Altogether, single-cell RT-qPCR data processing can, in general, still be considered straightforward compared with the other single-cell transcriptomics tools. The processed data can often be further analysed by either univariate methods (with necessary corrections for multiple testing), or multivariate analyses, such as hierarchical clustering or principal component analysis. In addition, more specialized probabilistic methods have been proposed[51].

## Global measurements of gene expression in single cells

RT-qPCR has several advantages but is limited to relatively small numbers of genes and is impractical to scale above a certain level, even with advanced microfluidic devices. To perform single-cell transcriptome analysis on a global scale, one can use microarray or RNA-seq technologies (Table 1). So far, these methods have mostly been used to screen for candidate genes that are subsequently validated with other methods such as RT-qPCR, flow cytometry or single-molecule fluorescence in situ hybridization (FISH) [45, 47, 52–54]. Each single-cell transcriptomic assay experiment, regardless whether is using microarrays or sequencing, can be divided into the following steps: (1) isolation of single cells, (2) cell lysis, (3) reverse transcription, (4) amplification of cDNA, (5) preparation of sequencing libraries and (6) eventually detection.

### Single-cell isolation

The first and sometimes underappreciated step is to isolate single cells. Whereas many immune cell types naturally exist as single-cell suspensions, other cells have to be dissociated from the tissue. Such treatment is far from trivial, as it requires enzymatic or mechanical approaches that may affect not only the intactness and viability of cells but also their transcriptomes.

Historically, in the first single-cell mRNA experiments, single cells were manually selected and picked from the early embryo using micropipetting [17, 55, 56]. The advantage of this approach is that particular cells of interest can be selected and cell losses can be minimized in the process. Suspended single cells can be sorted into wells of a microtitre plate using fluorescence activated cell sorting (FACS) [57], they can be separated using microfluidic devices such as the Fluidigm C1^TM^ [23, 47, 58–61] or they can be encapsulated in nanolitre droplets (Table 2) [11, 62].

**Table 2.** Comparison of scRNA-seq platforms

Characteristic	FACS	Microfluidics (e.g. Fluidigm C1^TM^)	Droplets (DropSeq, InDrop, Chromium^TM^, etc.)
Reaction volume	Microliter	Nanoliter	Nanoliter
Throughput	Low to medium (depends on level of automatization of RT, amplification and library preparation processes)	Low to medium (depends on chip design)	High (limited by number of distinct cell barcodes)
Flexibility	Great flexibility to choose methods for RT, amplification and library preparation	Some flexibility to choose methods for RT, amplification and library preparation	Only molecule counting (no full-length transcript coverage)
Additional measurements	Additional data from index sorting (size, granularity, expression of surface markers, DNA content, etc.)	Imaging of the captured cells before lysis	None

The key advantage of FACS is the possibility to sort for particular subpopulations using molecular markers. In addition, the intensity of the fluorescence of several fluorescent markers along with values of forward and side scatter can be recorded for each cell. This provides useful phenotypic information about protein abundance, cell size and granularity on top of the single-cell transcriptomes [63]. When studying known, rare cell types (e.g. blood stem cells), FACS can capture essentially all cells from the population of interest. The main disadvantage of using FACS to sort single cells into microtitre plates are the microlitre reagent volumes involved, which can be prohibitively expensive in large-scale experiments as compared with nanolitre volumes involved in microfluidics and droplet-based methods [64]. The Fluidigm C1^TM^ is a microfluidic platform that captures single cells (96 or 800 cells per chip) and performs reverse transcription and amplification of cDNA by PCR on chip. As all these reactions are carried out in nanolitre volumes, this leads to lower reagent costs. Importantly, this platform enables microscopic inspection of each cell on capture, which allows identification of positions where multiple cells or debris were captured. A drawback of the C1^TM^ workflow is the relatively low capture efficiency. To capture 96 cells on C1 ^TM^, one typically requires a starting population of at least 1000 cells, making the method impractical for rare populations. Another important limitation of this method is that cells being captured have to be homogeneous in size and compatible with one of the available capture site sizes (5–10, 10–17 and 17–25 µm in diameter). Nonspherical or sticky cells also do not capture well, but at the same time, this capture method is much more gentle than FACS, and hence is suited to delicate cell types such as neurons, megakaryocytes, etc.

Recently, droplet-based microfluidics methods have been published, namely, inDrop [62], Drop-Seq [11] followed by launching of similar commercial protocols such as the Chromium^TM^ from 10X Genomics [65]. These protocols encapsulate single cells, or single cells and beads bearing barcodes, in aqueous droplets within a surrounding oil phase. The droplets can be subsequently fused with other droplets to deliver reagents to perform lysis, reverse transcription and PCR. Reagent can also be delivered into droplets using picoinjection [66]. These methods will likely prove especially useful for surveying cells from different tissues to identify new cell types and cell functions, as they allow analysis of several thousands of cells in one experiment.

Less frequently used methods include laser capture microdissection, which is useful for picking cells from a particular position in a tissue. It is low throughput and does not necessarily guarantee that a single cell, rather than small group of cells is captured [67, 68]. Finally, nanolitre plates can be used for capturing single cells. Simply by adjusting the concentration of the cells in suspension, cells can be deposited and virtually every well will receive zero or one cell [69, 70].

### Cell lysis

Captured cells are lysed by addition of lysis buffer containing detergent to disrupt the cell membrane. For plant or fungi cells, protoplasts must first be obtained by enzymatic or mechanical removal of the cell wall. Efficient cell lysis is crucial for efficient release of RNAs to the reaction and for the efficiency of subsequent reactions.

### Reverse transcription

In the next step, RNAs are reverse transcribed, and this is a key step for achieving high sensitivity. A major goal of this stage is to avoid reverse transcribing ribosomal RNAs (rRNAs), which are high-abundance and would dominate any signal from the much lower abundance mRNAs. Owing to the low abundance of mRNAs, common mRNA purification methods cannot be used. Most protocols for reverse transcription (SmartSeq [53], STRT-Seq [54], QuartzSeq [71]) use poly(T) primers that bind to the poly(A) tail of mRNAs. This way only polyadenylated RNA species are reverse transcribed.

Alternatively, primers that are specifically designed not to bind to rRNAs can be used 

[72]. The disadvantage of this approach is that it may lead to amplification biases against some mRNAs. Finally, it was shown recently that random hexamer primers can be used [73, 74], provided reverse transcription is performed at low temperature. In such conditions, most rRNAs are within folded ribosomes and are not transcribed. Moving beyond poly(A) priming would be useful for analyses of non-coding RNAs (ncRNAs), such as circular RNAs [74], and also bacterial RNAs, which are not polyadenylated [75].

Second-strand cDNA synthesis can be done using the template-switching properties of the reverse transcriptase to minimize detection of partially transcribed species: this approach is used in SmartSeq [53]. Alternatively, poly(A) tailing and subsequent second-strand synthesis priming from the polyA sequence can be used, but this leads to stronger 3′ bias of read coverage over transcripts, meaning that there are more reads mapping to the 3′ end of the transcript. This originates from incomplete reverse transcription, as in the first single-cell sequencing protocol by Tang and colleagues and the QuartzSeq protocol [55, 71].

It is estimated that a single cell contains around 10 pg of mRNA [53], which will not produce sufficient cDNA for sequencing library preparation alone; thus, the cDNA must be amplified. There are two main methods of amplification: linear amplification using IVT and exponential amplification using PCR. Most protocols use PCR for amplification: SmartSeq [53], SmartSeq2 [76], STRT [54], the Tang protocol [55] and SC3-seq [77]. The main caveat of PCR is the fact that the exponential amplification that occurs may distort the relative amounts mRNA molecules. The alternative approach of IVT was incorporated into the CEL-Seq [78], CEL-seq2 [79] and MARS-Seq [64] protocols. Amplification via IVT is linear, but it was shown that subsequent IVT causes significant shortening of amplified RNAs and thus only the 3′ ends of mRNAs are amplified [80].

The number of molecules in each cell is limited and it is estimated that only 10% of them are transcribed to cDNA with current technologies [81]. The molecules that are transcribed are selected stochastically. Owing to Poisson sampling, the expression-level estimation may not represent the original set of molecules from the cell, especially for lowly abundant mRNA species leading to so-called ‘drop outs’. Computational approaches are being used to alleviate their effects [82, 83].

### Library preparation and detection

Microarrays were initially used for detection of amplified cDNA [71, 84–90], but as they have lower robustness, low sensitivity, limited dynamic range and require large amount of cDNA for hybridization, they are now completely replaced by sequencing for the single-cell transcriptomic applications [91, 92].

Sequencing libraries are prepared from amplified cDNA using the same protocols as for conventional bulk mRNA sequencing experiments and can be sequenced on any sequencing platform. Both SOLID and standard Illumina library preparation protocols, involving Covaris shearing, ligation of adapters and library amplification, were used, but the most common is the Nextera^TM^ kit from Illumina that uses enzymatic Tn5-mediated tagmentation as well as home-brew version of this kit [53].

All RNA-seq methods allow multiplexing with barcoded adapters at the stage of library preparation. This means that barcoded adaptors can be ligated to the cDNA that results from preamplification. Both the standard library preparation kit and the Nextera^TM^ kit from Illumina and library preparation kits for SOLID^TM^ system have barcoding options. Barcoding before the stage of library preparation allows pooling samples to cut down costs of reagents and dramatically reduces sample handling. The STRT method, as well as currently used droplet methods depend on primers containing cell-specific barcodes that are introduced at the reverse transcription step of the protocol [78]. Similarly in CEL-Seq and CEL-Seq2 barcodes are introduced during IVT stage [79].

## Single-cell experiments require internal controls

Single-cell RNA-seq presents challenges that are absent in conventional population-level approaches. Distinguishing biological from technical variation in situation where technical replications are difficult to perform, as there are no two identical cells, is challenging. Furthermore, the sensitivity of the protocols is limited, which leads to so-called ‘drop-outs’, i.e. false-negative values for mRNAs that are present in low amounts but are not being detected. Thus, it is important to measure technical variation to understand which genes can be quantified accurately, and to minimize the rate of false positives in differential expression analysis.

Technical noise can arise at each step of the protocol, including differences in lysis efficiency and reverse transcription and the tendency of some species of mRNA to be preferentially amplified due to their sequence and length of their poly(A) tails. These biases have not yet been sufficiently systematically investigated. Secondly, there is variation in the measurement from batch to batch. This may be because of differences between operators, batches of reagents or other factors. Thirdly, single-cell RNA-seq data have the same biases as conventional RNA-seq, such as PCR amplification bias, sequence bias during fragmentation and coverage biases. Importantly, more rounds of amplification are required than in bulk RNA-seq providing more opportunities for the introduction of base substitutions. If amplification is performed using PCR, then PCR amplification biases are also present. It was also reported that reverse transcription with poly-dT priming leads to 3′ bias in read coverage [53, 93]. This is also the case in bulk-level experiment that uses poly-dT priming.

Technical variation between cells can be estimated using mRNA spike-ins that undergo all the steps of the protocol together with the sample. In early microarray experiments, a set of four *Bacillus subtilis* mRNAs (Lys, Dap, Phe and Thr) spiked-in at different copy numbers has been used to measure detection limits. ERCC (External RNA Control Consortium) spike-in is the most commonly used, commercially available set of control molecules and consists of 92 synthetic polyadenylated mRNA species of different known concentrations [94]. These were designed so as to lack sequence similarity to any known eukaryotic genome. It allows one to measure the sensitivity and accuracy of each experiment, as well as perform correction of some batch effects. It is also used for estimation of the extent of technical noise [95].

ERCC spike-ins can be used to produce a calibration curve to estimate the absolute number of molecules in each cell [85, 86, 96]. It should be noted that ERCC molecules do not go through cell lysis and are not associated with proteins, thus are not subjected to all the processes that cellular mRNAs are. Furthermore, they are not capped, they have short poly(A) tails in comparison with endogenous mRNAs and because of their easy degradation during normal handling, it is difficult to ensure accurate input concentrations [97]. SIRVs (Spike-In RNA Variant Mixes, Lexogen) are an alternative or complementary to ERCCs spike-in mix. They are designed to in addition to abundance control splicing patterns of RNAs, i.e. the spike-in consists of 69 different transcripts that mimic splice variants of 7 genes.

Interestingly, one can also use minute amounts of total RNA coming from a species alien to the species of interest as a spike-in. This approach provides thousands of technical data points across the whole dynamic range of expression, thereby ensuring that technical noise levels can be well quantified across the whole dynamic range [95]. The drawback of this approach is that a substantial number of reads goes to technical noise control, entailing significant costs. Technical variability within an experiment can be also estimated by performing pool and split experiments [98, 99].

**Figure 2.**
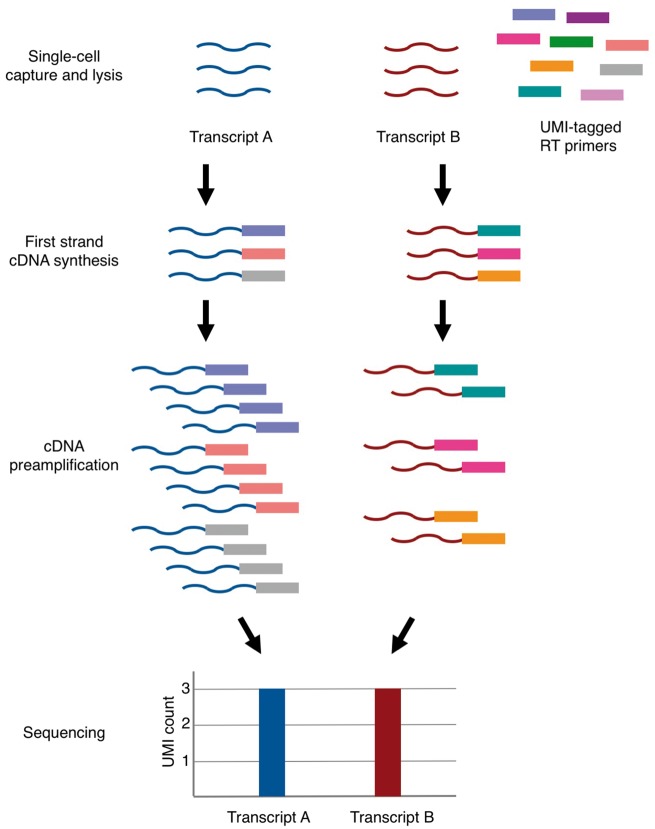
Molecular counting with UMIs. UMIs are random *n*-mer oligonucleotide sequences included in the reverse transcription primers. As the number of different UMI sequences exceeds the number of copies for any single-transcript species, the UMI sequences can be used for quantifying the number of molecules that were successfully captured and amplified, and thus control for amplification biases associated with PCR-based sample preparation.

While the use of spike-in RNA is relatively commonplace with protocols based on cell sorting or microfluidic cell capture devices, this strategy is less frequently used in droplet-based workflows. One limitation is that the spike-in molecules will be deposited also in partitions that do not contain cells, and therefore unnecessarily consume sequencing capacity. In addition, reads from such empty droplets might hinder detection of true single cells from the data. Instead of spike-in RNA, droplet-based workflows typically incorporate unique molecular identifiers (UMIs), which are highly diverse, random, unique barcodes for tagging each cDNA molecule generated during reverse transcription [54, 81, 100, 101]. They enable one to count molecules by counting the number of unique UMI sequences associated with each transcript instead of counting the number of sequencing reads that map to a particular transcript (Figure 2). This can ameliorate PCR biases [96]. The main disadvantage of UMIs is that until now they have only been used for methods that count the 3′ end of molecules. In addition, to estimate the number of molecules, one has to sequence deeply.

## Choosing the right protocol depends on the biological system under investigation

The optimal single-cell RNA-seq application depends on the desired application. Each of the single-cell methods described above has advantages and disadvantages as summarized in Table 1. Factors to be considered include throughput, sensitivity and robustness, transcript coverage, cost and handling (comprehensive sensitivity and robustness comparison of methods were performed by [97]). For example, for discovery of new cell types, tag-counting droplet methods with high throughput are most advisable, while for analysis of allelic expression or splicing, one must use a protocol that provides sequencing coverage of the entire length of mRNA molecules.

## Future outlook

Single-cell transcriptomics brings both new opportunities and new challenges. Measuring gene expression at the single-cell level provides a huge amount of information, which requires adequate data analysis methods. In past couple of years, several different approaches for analysis of single-cell sequencing data emerged and still new computational methods are being developed to access even more information from single-cell data [102, 103].

The current efforts of many groups are focused on approaches for ordering cells along a process in so-called ‘pseudotime’ to describe transitions between cell states and cellular decision points, where cells commit on one of available states [104–110]. As single-cell data have and even with improvement of technology will inevitably suffer from false negatives because of dropout effect, computational approaches to include this effect into models and analysis are crucial [83].

Furthermore, there is room for improvement of experimental side of single-cell methods. One issue that should be addressed is sensitivity and robustness, with more efficient chemistry and RNAse-free reagents we may be able to detect more genes and limit the dropouts. Secondly, sample size, i.e. the number of single cells sequenced is crucial to obtain statistical power and to observe rare cell types. Further developments are needed to increase throughput [111, 112], simultaneously allowing multiplexing different biological samples in one run [113]. Thirdly, further developments are needed to streamline single-cell sequencing of non-polyadenylated RNA species, to detect bacterial RNA as well as eukaryotic ncRNAs and combine it with other measurements in the same cell, such as imaging, genome and epigenome analysis or protein abundance quantification [114].

Finally, it is important to bear in mind that single-cell experiments, though informative and can help elucidate many crucial biological problems, are only part of the equation. Localization of mRNA is as important as its abundance; hence, there is a lot of effort to develop protocols that retain spatial information about the transcripts (TIVA [115], FISSEQ [116, 117] or padlock probe-based methods [118]. Cells are part of complex tissues and interact with each other both physically and by using different chemical signals; thus, understanding single cells in the context of complex tissues will be the next challenge for single-cell research.

**Key Points**

- Studies of cell population heterogeneity and cell transitions benefit from single-cell approaches.
- Single-cell quantitative PCR allows study of gene expression heterogeneity in population of cells.
- Single-cell RNA-seq experimental approach can be chosen depending on needed number of cell, gene detection efficiency, transcript coverage, cost, etc.
- Technical biases and artefacts are determined using spike-ins and UMIs.
- Spatial transcriptomics and combination of RNA quantification with other measurements from a single cell are next steps in the field.

## Funding

Tapio Lönnberg is supported by the Academy of Finland (Decision 311081).

## Notes

**Aleksandra A. Kolodziejczyk** studies single cells using RNA sequencing since 2012, when she started her PhD in the laboratory of Sarah Teichmann Wellcome Trust Sanger Institute and EMBL European Bioinformatics Institute.

## Notes

**Tapio Lönnberg** was familiarized with single-cell transcriptomics during his postdoctoral work with Sarah Teichmann at the EMBL European Bioinformatics Institute and the Wellcome Trust Sanger Institute. He is currently an Academy of Finland postdoctoral fellow at the University of Turku, Finland.
